# Natural killer cells efficiently target multiple myeloma clonogenic tumor cells

**DOI:** 10.1007/s00262-021-02901-y

**Published:** 2021-03-10

**Authors:** Alejandra Leivas, Ruth M. Risueño, Alma Guzmán, Laura Sánchez-Vega, Manuel Pérez, Diego Megías, Lucía Fernández, Rafael Alonso, Antonio Pérez-Martínez, Inmaculada Rapado, Joaquín Martínez-López

**Affiliations:** 1 Hematology Department, Hospital Universitario 12 de Octubre, Complutense University, Instituto de Investigación Sanitaria Hospital 12 de Octubre (imas12), Madrid, Spain; 2 H12O-CNIO Haematological Malignancies Clinical Research Unit, Spanish National Cancer Research Center, Madrid, Spain; 3 Leukemia Stem Cell Group, Josep Carreras Leukaemia Research Institute, Barcelona, Spain; 4 Confocal Microscopy Unit, Spanish National Cancer Research Center, Madrid, Spain; 5 Pediatric Hemato-Oncology Department, Hospital Universitario La Paz, Madrid, Spain

**Keywords:** Side population, Stem cell, Clonogenic cell, NK cells, Cell therapy

## Abstract

The multiple myeloma (MM) landscape has changed in the last few years, but most patients eventually relapse because current treatment modalities do not target clonogenic stem cells, which are drug-resistant and can self-renew. We hypothesized that side population (SP) cells represent myeloma clonogenic stem cells and, searching for new treatment strategies, analyzed the anti-myeloma activity of natural killer (NK) cells against clonogenic cells. Activated and expanded NK cells (NKAE) products were obtained by co-culturing NK cells from MM patients with K562-mb15-41BBL cell line and characterized by flow cytometry. Functional experiments against MM cells were performed by Eu-TDA release assays and methylcellulose clonogenic assays. Side population was detected by Dye Cycle Violet labeling and then characterized by flow cytometry and RNA-Seq. Self-renewal capacity was tested by clonogenic assays. Sorting of both kind of cells was performed for time-lapse microscopy experiments. SP cells exhibited self-renewal potential and overexpressed genes involved in stem cell metabolism. NK cells from MM patients exhibited dysregulation and had lower anti-tumor potential against clonogenic cells than healthy donors’ NK cells. Patients’ NK cells were activated and expanded. These cells recovered cytotoxic activity and could specifically destroy clonogenic myeloma cells. They also had a highly cytotoxic phenotype expressing NKG2D receptor. Blocking NKG2D receptor decreased NK cell activity against clonogenic myeloma cells, and activated NK cells were able to destroy SP cells, which expressed NKG2D ligands. SP cells could represent the stem cell compartment in MM. This is the first report describing NK cell activity against myeloma clonogenic cells.

**Supplementary Information:**

The online version of this article (10.1007/s00262-021-02901-y) contains supplementary material, which is available to authorized users.

## Introduction

The treatment landscape for multiple myeloma (MM) has changed in the last few decades with remarkable improvement of median survival [1]. However, most MM patients eventually relapse and the disease remains incurable. Recent studies have revealed that MM clonogenic stem cells (CSCs) play a critical role in disease resistance to both radiation and chemotherapy [2]. CSCs are significantly less sensitive to current therapies than non-clonogenic cells [3]. After treatment, MM CSCs are able to replenish the bulk of the tumor, leading to disease relapse [4, 5].

Several methods for identifying CSCs have been described [6, 7], of which side population (SP) detection is the most reliable method. This population was first described as a subset of adult mouse bone marrow cells enriched with hematopoietic stem cells [8]. SP cells are a minority population characterized by an ability to efflux DNA binding-dyes, such as Hoechst 33,342 or Dye Cycle Violet (DCV), via an ATP-binding cassette transporter; they have a low dye-staining profile on flow cytometry [9]. Therefore, SP detection is directly related to resistance to chemotherapy and disease relapse. Recent studies have shown the presence of SP cells in many types of cancer, including gastrointestinal cancers, lung cancer, and ovarian cancer. In these types of cancer, SP cells exhibit higher potential to initiate tumors in NOD/SCID mice than non-SP cells [10, 11]. In MM, SP cells transplanted into NOD/SCID mice exhibit a capacity for tumor formation [12].

The interaction between malignant cells and immune cells is critical in determining disease outcome in cancer patients. In MM, SP cells are able to grow and spread into the bone marrow due to impaired cancer immunosurveillance and the development of immune escape mechanisms [13]. The functionality of immune cells, such as T cells and natural killer (NK) cells, is compromised in MM patients, resulting in defective antigen presentation and poor NK cell capacity to kill transformed or infected cells and tumor cells [14]. NK cells recognize and destroy infected or transformed cells very quickly without prior sensitization; therefore, the activation of NK cell effector functions may represent a promising therapeutic strategy that could lead to better immunosurveillance in MM patients [15].

Several protocols for activation and ex vivo NK cell expansion have been investigated [16, 17]. We recently showed that NK cells from refractory MM patients can be activated and expanded by co-culturing with K562-mb15-41BBL cells in the setting of a phase I clinical trial (NCT02481934). These activated and expanded NK (NKAE) cells exhibit potent cytotoxic activity against bulk myeloma cells. Moreover, we demonstrated that these NKAE cells in combination with anti-myeloma drugs have clinical efficacy in MM patients [18].

To date, the evidence suggests that most of the current treatments do not target MM CSCs [19]. NKAE cells could be a promising therapeutic option for MM, but the ability of NKAE cells to destroy CSCs remains unknown. Here, we address this issue and demonstrate that primary NK cells could destroy MM CSCs.

## Materials and methods

### Human samples and cell lines

Fresh peripheral blood (PB) samples from MM patients and healthy donors were used. Fresh bone marrow samples from patients with newly diagnosed MM or relapse were used.

K562-mb15-41BBL cells were kindly provided by Dario Campana, former researcher from St. Jude Children’s Research Hospital (Memphis, TN). U-266, L-363, JJN-3, and OPM-2 myeloma cells were purchased from DSMZ (Braunschweig, Germany). Cells were incubated in RPMI-1640 medium (Biowest, Nuaillé, France) with 10% fetal bovine serum (FBS, Hyclone, GE Healthcare, Little Chalfont, United Kingdom) in a humidified 5% CO_2_ chamber at 37 °C.

### NK cell activation and expansion

PB mononuclear cells (PBMCs) were isolated and then activated and expanded for 3 weeks to achieve NKAE expansion. The cells were co-cultured with the K562-mb15-41BBL feeder cell line plus 100 IU/mL IL-2 as described previously [20, 21].

### Flow cytometric analysis and fluorescence-activated cell sorting

NK and NKAE cells (CD3−CD56+) were analyzed by flow cytometry based on the percentage of the NK cell population and expression of activating receptors NKG2D and NKp30. The gating strategy was based on dead/live cells and doublet discrimination. The antigen expression profiles of NK and NKAE cells were evaluated using the following fluorescence-conjugated antibodies: CD69, CD25, CD31, DNAM-1, FasL, CD7, TRAIL, NKG2D, NKp46, NKp30, NKp44, and NKG2A (Supplementary Table 1). The data were analyzed by FACSDiva™ software (BD Biosciences San Jose, CA, USA). NK cells were purified to perform time lapse microscopy assays. To avoid collateral effects from antibody labeling on NK cell cytotoxicity, NK cells were sorted by CD3^+^, CD19^+^, and CD14^+^ cell exclusion on a three laser BD FACS Aria™ Fusion cell sorter (BD Biosciences).

### Functional assays

The cytotoxicity of PBMCs and NKAE cells was assessed as described previously [18, 21] using Eu-TDA release assays following the manufacturer’s instructions. U-266, L-363, and OPM-2 MM cells were used as target cells and incubated for 2 h with effector cells (PBMCs or NKAE cells) at the indicated effector:target (E:T) ratio.

Colony forming assays were performed to evaluate NK cell cytotoxicity against clonogenic myeloma cells [22]. MM cells were co-cultured with NK cells at E:T ratios of 32:1, 16:1, 8:1, and 4:1 E:T or cultured alone (control) for 2 h at 37 °C and in a 5% CO_2_ atmosphere. Cells were then suspended in methylcellulose (Methocult, Stem Cell Technologies, Vancouver, Canada) and seeded in triplicate in a humid chamber to avoid methylcellulose dehydration. Plates were incubated for 14 days and colonies counted. Representative images were acquired in a G:BOX Chemi XX6 transiluminator (Syngene, Synoptics, Cambridge, United Kingdom). GeneSys image acquisition software was used for analysis (Syngene). To rule out NKAE cells causing hematological toxicity, colony forming assays against healthy CD34^+^ progenitor cells were performed.

NKG2D (clone 1D11, Biolegend, San Diego, CA, USA) and NKp30 (clone P30-15, Biolegend) receptor blocking assays were performed using purified saturating concentrations of monoclonal antibodies (10 µg/ml and 5 µg/ml, respectively) as described previously [23].

### Side population

SP of MM cells was evidenced by DCV staining (5 μM, Thermo Fisher Scientific, Waltham, MA, USA) for 90 min at 37 °C in the absence or presence of the ABC transporter inhibitor reserpine (50 μM, Sigma-Aldrich, St. Louis, MO, USA). SP analysis of MM cells was performed by flow cytometry. Fresh bone marrow samples were also stained with CD138-FITC (Biolegend, San Diego, CA, USA) and CD38-PE/Cy7 (Biolegend, San Diego, CA, USA) to identify MM plasma cells. DCV was excited at 405 nm and its emission fluorescence detected using 450/50 nm (DCV blue) and 675/20 (DCV red) band pass filter systems. To determine if SP cells exhibit clonogenic properties, colony forming assays were also performed after sorting of SP cells. SP cells were seeded in triplicate at 300 cells/ml and incubated as described above.

### Immunofluorescence

MM cells from the RPMI-8226 MM cell line and NKAE cells were incubated at 37 °C for 15 min at a ratio of 1:2 (NK:MM). Cells were fixed and permeabilized, incubated with Image-IT FX Signal Enhancer (Thermo Fisher Scientific, Waltham, MA, USA) and human γ globulin (100 μg/ml, Sigma-Aldrich, St. Louis, MO, USA). Then, cells were stained for 30 min with primary antibodies against NKG2D or NKp30 receptors (1:200 dilution) at 37 °C in a 5% CO2 atmosphere and, then, with secondary Alexa Fluor 488 antibody (1:200, Thermo Fisher Scientific) and DAPI (0.5 μg/ml, Sigma-Aldrich). Cells were analyzed using an SP5 confocal microscope (Leica Microsystems CMS GmbH, Wetzlar, Germany).

### Time-lapse microscopy

MM cells (bulk MM cells or SP cells) from RPMI-8226 cell line were seeded on a six-channel polymer-treated flow chamber at 1 × 10^6^ cells/ml, forming a monolayer, and incubated overnight in a humidified chamber at 37 °C and 5% CO_2_ in RPMI-1640 medium containing 10% fetal bovine serum (FBS) and penicillin/streptomycin (Thermo Fisher Scientific, Waltham, MA, USA) to favor cell adherence. NK or NKAE cells were resuspended at 0.5 × 10^6^ cells/ml in RPMI medium containing 10% FBS and antibiotics. Channels with adhered MM cells were washed for 5 min with PBS before the establishment of NK cell flux. After NK cell flux, a 2-h flux of RPMI medium was performed to evaluate MM cell death. MM cell death was quantified in four different positions for each channel. Cells were analyzed using a Leica AF6000W microscope.

### RNA isolation and RNA-Seq

Total RNA was extracted according to the manufacturer’s protocol (Qiagen, Hilden, Germany). RNA quantity and quality were assessed by 2100 Bioanalyzer (Agilent Technologies, Santa Clara, CA, USA). The ribosomic RNA was depleted (Thermo Fisher Scientific) according to the manufacturer’s instructions. RNA was fragmented by incubation with RNase III for 3 min at 37 °C. RNA sequencing libraries were constructed following the manufacturer’s recommendations using an Ion Total RNA-Seq Kit (Thermo Fisher Scientific).

The quality of the amplified libraries was verified using an Agilent Technologies 2100 Bioanalyzer. Clonal amplification and chip loading (Ion PI Hi-Q Sequencing 200 Kit and Ion PI Chip v3, Thermo Fisher Scientific) were carried out on the Ion Chef System (Thermo Fisher Scientific), and then the chips were loaded on an Ion Proton sequencing system (Thermo Fisher Scientific). High quality clean reads mapped to the hg19 using a combination of TopHat2 (ver. 2.3.1j) and Bowtie (ver.2.2.1.0) alignment. Enrichment analysis was performed based on Gene Ontology (GO) Consortium.

### Statistical analysis

Normal distribution of the population and homocedasticity were evaluated. Then, assay data were compared using Student’s *t* test. Clonogenic assays data were compared using ANOVA. Differences between groups were analyzed using Bonferroni post-hoc test. Non-parametric tests were applied for samples that do not follow a normal distribution. Significance was defined as *p* ≤ 0.05. Analyses were performed using SPSS v.23 and GraphPad Prism v8 was used for graphical representation. Differentially expressed genes from RNA-Seq were quantified by gene-specific analysis (GSA, Genomics Suite Software, Partek, St. Louis, MO, USA). For GSA, a false discovery rate (FDR) < 0.05 and twofold change were used to consider relevant differences. Enrichment analysis was performed based on Gene Ontology (GO) Consortium. GO terms with a fold change > 2 and p < 0.05 were considered functionally relevant.

## Results

### Side population identification

**Fig. 1:**
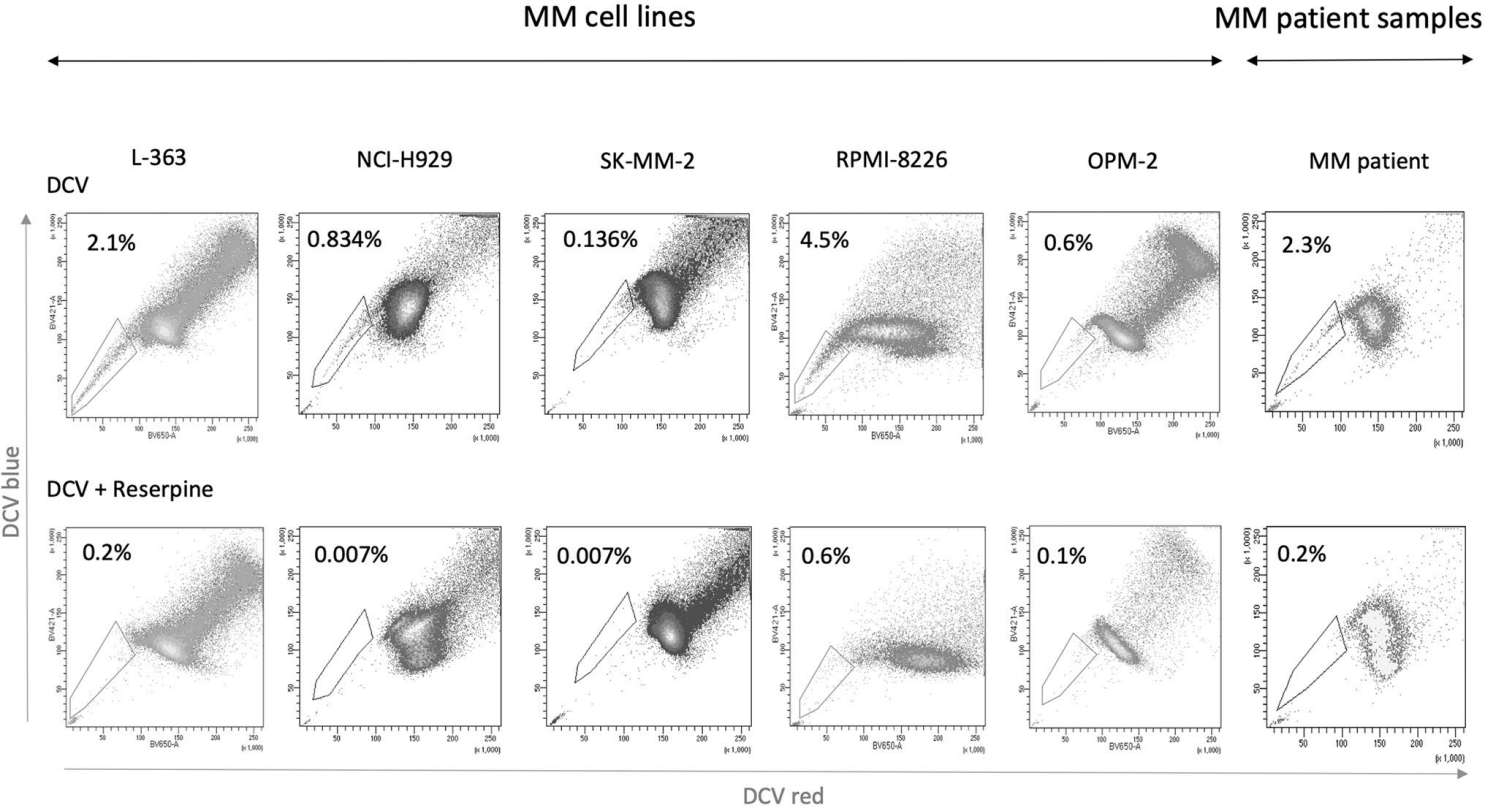
Identification of the MM side population (SP). **a** Representative flow cytometry dot plots for the SP from cell lines (top) and primary samples (top left). The SP disappeared when cells were treated with reserpine (bottom)

SP cells were identified among MM cells from eight distinct MM cell lines (RPMI-8226, U-266, L-363, JJN-3, OPM-2, NCI-H929, SK-MM-2, and MM.1S) and eight fresh primary bone marrow samples from MM patients (Fig. 1 and Supplementary Figure S1, Supplementary File 1). The SP was present in all cell lines, but the percentages of SP varied from one cell line in the range of 0.2%–11.9% (Fig. 1 and Supplementary Table S3, Supplementary File 1). Flow cytometry also revealed the presence of SP cells in MM plasma cells from bone marrow samples (Fig. 1 and Supplementary Table S2, Supplementary File 1), varying from 0.2 to 11.48%. The SP disappeared when the cells were incubated with reserpine in both types of samples (Supplementary Table S3, Supplementary File 1). The SP was also identified in one sample of plasma cell leukemia (PCL), with the SP representing 50% of the total pathological plasma cells from peripheral blood (data not shown).

### Side population from MM exhibit stem cell properties

**Fig. 2:**
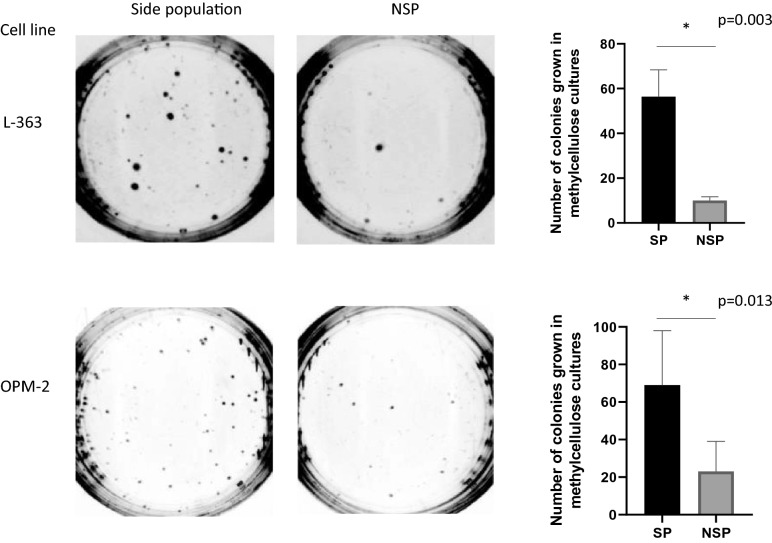
Stemness of side population (SP) cells. Clonogenic properties of SP cells. SP and non-SP (NSP) cells from two distinct MM cell lines were sorted and seeded at 300 cells/ml on serum-free methylcellulose. After 14 days of culture, clonogenic cell colonies were counted. Results are represented as mean ± SEM. Unpaired *T* test was performed (*p value < 0.05) to compare the number of SP and NSP colonies of each MM cell line (L-363 or OPM-2)

SP and non-SP cells from MM cell lines were sorted and seeded on methylcellulose dishes to determine the in vitro colony formation potential of these populations. SP cells were able to successfully grow on methylcellulose, requiring only 300 cells/plate, whereas non-SP cells had less replicative potential (Fig. 2). After 14 days in culture, SP cells from the L-363 cell line generated approximately 6-times as many non-SP colonies, and SP cells from the OPM-2 cell line generated approximately 3-times as many non-SP colonies. These findings corroborate the SP having a stronger self-renewal capacity than non-SP cells.

**Fig. 3:**
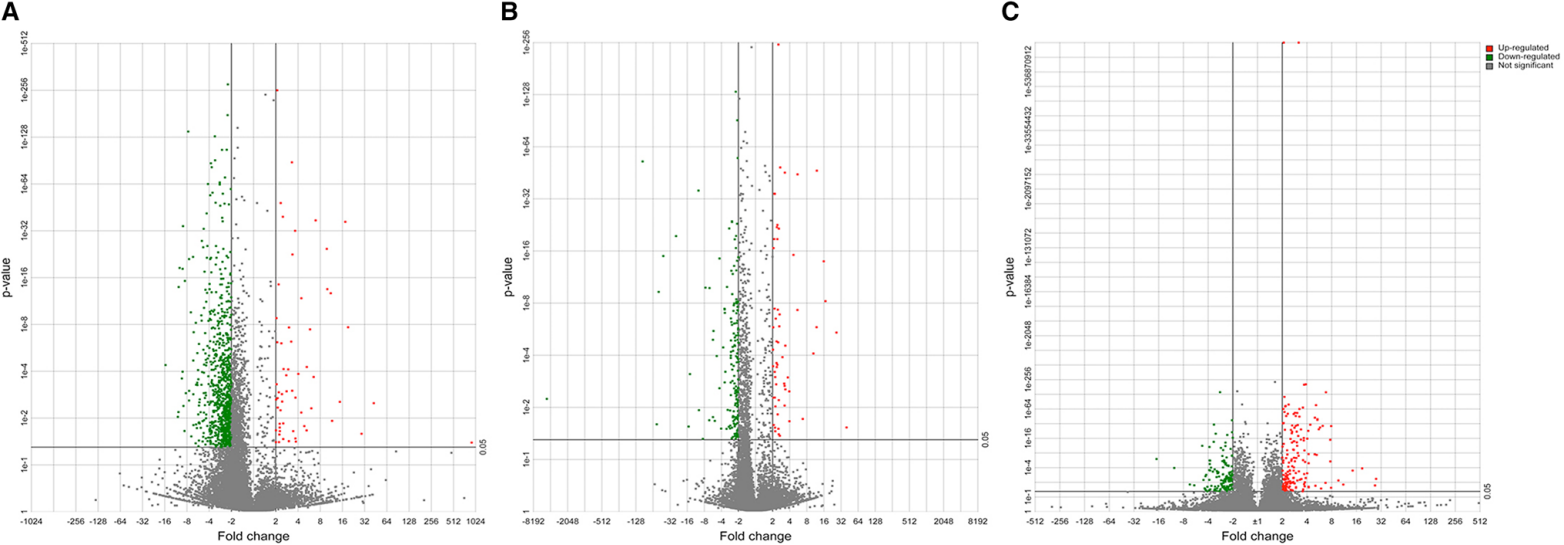
Gene-specific analysis (GSA) of the SP and NSP from primary MM samples. Three primary samples from **a** MGUS, **b** MM and **c** PCL were analyzed. Results from the different samples are represented as volcano plots. A *p* value < 0.05 and twofold change were used to consider relevant statistical differences in gene expression

RNA-Seq experiments were performed with two MM cell lines (L-363 and OPM-2) and three primary samples: one monoclonal gammopathy of undetermined significance (MGUS), one MM, and one PCL. Genes involved in the negative regulation of stem cell differentiation and stem cell maintenance were overexpressed in MGUS, PCL, and OPM-2 SP cells compared to non-SP cells. MGUS cells also exhibited overexpression of three genes involved in histone methylation (GO IDs are summarized in the Table 1). Genes involved in cell division and proliferation were significantly overexpressed in the SP from all samples: 26 in MGUS, 7 in MM, 12 in PCL, 15 in OPM-2 cells, and 16 in L-363 cells. GSA revealed that PCL sample exhibited a greater number of upregulated genes in the SP than in the NSP when compared to MGUS or MM samples. However, the MGUS sample showed the opposite result, the NSP showed a greater number of upregulated genes. No differences were observed in terms of the number of upregulated genes between SP and NSP in MM sample (Fig. 3). Of note is the overexpression of small nucleolar RNA (snoRNA) encoding genes in the SP. Upregulated genes included a large number of genes encoding snoRNA, 70 snoRNAs were upregulated in the LCP sample. In the case of MM, there were 17 snoRNA upregulated genes and 14 in the MGUS sample (Dataset from RNA-seq can be found on a public repository, 10.17632/4nmjyxyb93.2).

**Table 1:** Gene ontology identification of genes involved in stem cell metabolism

Sample	Gene GO ID	Function	*p* value
MGUS	2000737	Negative regulation of stem cell differentiation	0.0378
MGUS	35019	Somatic stem cell maintenance	0.046
MGUS	51568	Histone H3-K4 methylation	0.009
MGUS	34968	Histone lysine methylation	0.018
MGUS	16571	Histone methylation	0.0426
MM	19827	Stem cell maintenance	0.006
PCL	17145	Stem cell division	0.0173
PCL	48863	Stem cell differentiation	0.0139
PCL	42078	Germ-line stem cell division	0.024
PCL	48133	Male germ-line stem cell asymmetric division	0.024
PCL	98722	Asymmetric stem cell division	0.024
PCL	98728	Germ-line stem cell asymmetric division	0.024
OPM-2	42078	Germ-line stem cell division	0.041
OPM-2	48133	Male germ-line stem cell asymmetric division	0.041
OPM-2	98722	Asymmetric stem cell division	0.041
OPM-2	98728	Germ- N7line stem cell asymmetric division	0.041
OPM-2	35019	Somatic stem cell maintenance	0.041
OPM-2	19827	Stem cell maintenance	0.0014

Genes overexpressed in the side population compared to the non-side population of different samples were classified according to their role in cell function, the biological process in which they are involved, and the cellular components in which they act. A Gene Ontology identification number was also provided

CD138 expression was evaluated because some authors have reported that MM stem cells are CD138-negative [24]. No differences in CD138 expression were detected between SP cells and non-SP cells from MM cell lines. However, SP cells from bone marrow samples exhibited higher, though variable, expression of CD138 than non-SP cells from bone marrow samples (Supplementary Figure S2, Supplementary File 1). These findings were corroborated by RNA-Seq analysis, in which MM and PCL SP samples overexpressed CD138 (FDR = 0.00187).

### NK cells target the side population

**Fig. 4:**
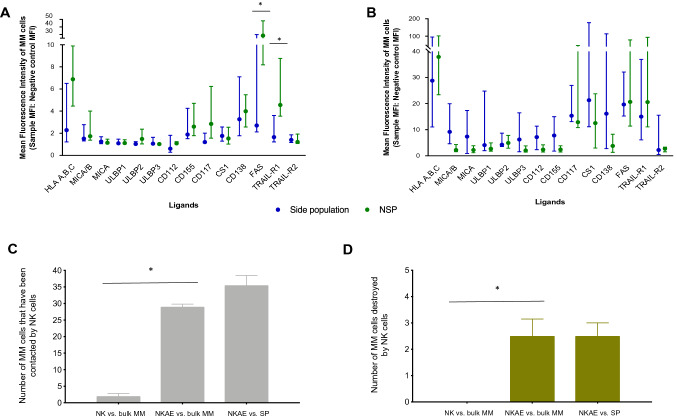
Side population (SP) sensitivity to NKAE cell activity. Expression of NK cell ligands in SP and non-SP (NSP) cell membranes from **a** eight MM cell lines and **b** eight primary MM cells from the bone marrow of MM patients. Data are presented as the mean fluorescence intensity (MFI) ratio. MFI ratio was defined as the MFI of the specific staining relative to the MFI of the appropriate fluorescence minus one control staining. *p < 0.05 compared to NSP cells. **c** Each NK cell contact with an MM cell was counted in the four fields of each channel during the 20 min of NK cell flux. **d** In addition, MM cells that underwent necrosis or apoptosis in each field were quantified. Data are represented as mean ± SEM of the four different positions for each condition. Unpaired T test was performed between NK cells and NKAE cells, and between NKAE vs. SP and NKAE vs. bulk MM. *p < 0.05 compared to NK cells (see Supplementary Video S1-S2, Supplementary File 2)

Expression of NK cell ligands, some of which were only expressed on tumor cells, was determined by flow cytometric analysis of SP cells. The SP cells expressed ligands for NK cell cytotoxicity receptors, including NKG2D. ULBP-1, -2, and -3, MICA, and MICB were expressed in SP cells (Fig. 4a, b), and the expression was not lower than in non-SP cells. In addition, DNAM-1 ligands (CD155 and CD112) and CS1 were expressed at similar levels in SP cells and non-SP cells (Fig. 4a). Moreover, the SP from primary MM samples (Fig. 4b) exhibited overexpression of NKG2D ligands, DNAM-1 ligands, and CS1. These data suggest that SP cells from MM could be targeted by NK cells.

Interestingly, SP cells from both MM cell lines and primary MM samples exhibited downregulation of apoptosis receptors, including FAS and TRAIL-R1 (Fig. 4a, b).

Time-lapse microscopy demonstrated that NKAE cells had better migration capacity than patients’ NK cells, as they had a greater ability to leave the cell flux and establish cell contacts with RPMI-8226 MM cells that adhered to the channel (Supplementary Video S1 in the linked Supplementary File S2). NK cell contacts and MM cells that underwent a necrotic or apoptotic process were counted during the experiment. NKAE cells were also able to establish many contacts with different MM cells during the experiment, though patients’ NK cells could not establish any contact at the same time and no MM cell underwent necrosis or apoptosis (*p* < 0.001, Fig. 4c, d). MM cells undergoing necrosis after NKAE cell contact are shown in Supplementary Figure S3 (Supplementary File S1). When NKAE cells were exposed to purified SP cells, no differences in NKAE cell cytotoxic activity were observed (*p* = 0.083). NKAE cells had the ability to contact and destroy the same number of MM cells, even if they were SP cells or bulk MM cells (Supplementary Video S2 in the related Supplementary File S2).

### NK cells destroy MM clonogenic tumor cells

**Fig. 5:**
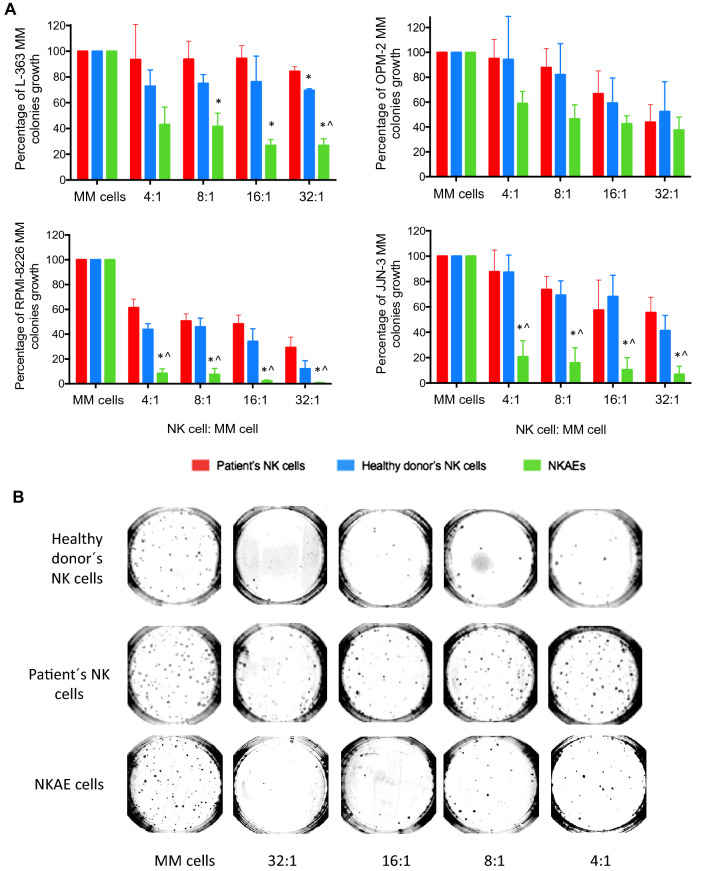
NKAE cells destroy clonogenic MM cells. Activity of NKAE cells against clonogenic tumor cells. **a** JJN-3, L-363, OPM-2, and RPMI-8226 cells were exposed to different NK cell concentrations (4:1, 8:1, 16:1, and 32:1). Data are presented as mean ± SEM of more than three independent experiments for each MM cell line and each NK cell concentration and are expressed as the percentage of MM colonies grown in methylcellulose after NK cell exposition and 14 days of culture. The reference was the number of MM cell colonies in the absence of NK cells. ANOVA test was performed between the different groups (patient´s NK cells, patients NKAE and healthy donor’s NK cells) for the same E:T ratio. *p < 0.05 compared to patient NK cells for the same NK:MM cell ratio, ^*p* < 0.05 compared to healthy donor NK cells for the same E:T ratio. **b** Representative images from one experiment of RPMI-8226 MM colonies from methylcellulose petri dish cultures after NK cell exposure and 14 days culture

NK cell cytotoxic activity against MM cells was evaluated by colony forming assays and Eu-TDA release assay. Colony forming assays allow quantification of cytotoxic activity against clonogenic tumor cells. In contrast, Eu-TDA release assays are intended to evaluate cytotoxicity against bulk MM cells. Activity against four different MM cell lines was analyzed (Fig. 5a). In general terms, the higher the concentration of NK cells in the culture, the fewer MM colonies are able to grow, which indicates a decrease in clonogenic tumor cells because they have previously been destroyed by NKs cells. When MM cells were exposed to NK cells from MM patients, fewer colonies were observed (Fig. 5b) in a dose-dependent manner, but they exhibited weak activity compared to those of NK cells from healthy donors and NKAE cells (Fig. 5a). These results suggest the presence of NK cell dysfunction in MM patients.

**Fig. 6:**
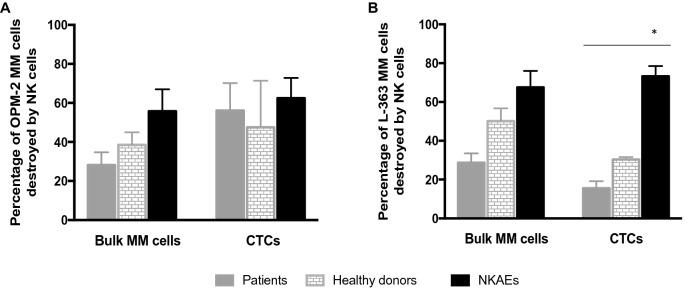
Destruction of bulk MM tumor cells and MM clonogenic cells from MM cells lines. Representative cytotoxic activity of NKAE cells from patients on L-363 (**a**) and OPM-2 (**b**) cells at a 32:1 ratio. Results are presented as mean ± SEM of a minimum of three independent experiments for each NK cell samples and for each kind of MM cell (total bulk MM cells or CTC) and are expressed as the percentage of MM cells destroyed compared to the autonomous growth of MM cells. ANOVA and post-hoc Bonferroni test were applied. *p < 0.05 NKAE vs. patient and healthy donor NK cells from the same group (L-363 CTCs)

NKAE cells overcame the impairment of patients’ NK cells and were able to destroy even more clonogenic MM cells even more than healthy donor NK cells (Fig. 5). This activity was highly variable depending on the MM cell line used as target. RPMI-8226 and JJN-3 cells were nearly completely destroyed by NKAEs (99.10% ± 0.2% and 93.2% ± 6.4%, respectively), whereas L-363 and OPM-2 cells were more resistant to NKAE cell activity (73.2% ± 5.3% and 63.4% ± 10.35%, respectively). The differences between NK cell activity against bulk cells and CTC cells revealed that NKs from MM patients are able to destroy a small percentage of bulk cells (which includes CTCs and non-clonogenic cells), but on the other hand, depending on the MM cell line used as target, this activity may be decreased (L-363 cells, Fig. 6b) or even increased (OPM-2 cells, Fig. 6a) specifically against CTCs. Even NK cell from healthy donors exhibited decreased cytotoxic activity against CTCs from L-363 cell line (Fig. 6b). In this sense, NKAEs overcome this impairment and destroyed both bulk and CTCs much more efficiently, and without any effect due to the cell line used as a target (Fig. 6).

**Fig. 7:**
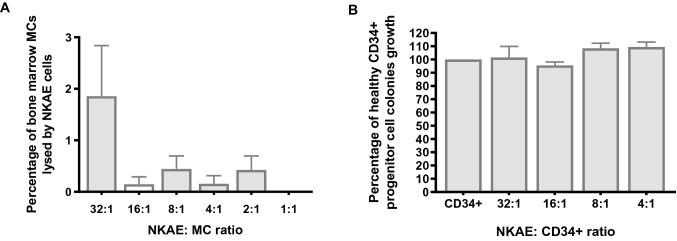
Effect of NKAE cell activity on hematopoietic cells. **a** To evaluate NKAE cell hematological toxicity on healthy tissue, bone marrow mononuclear cells (MCs) were exposed to different NKAE concentrations. Data are shown as the mean value ± SEM of four independent experiments. **b** Healthy CD34+ progenitors from healthy donor bone marrow samples were exposed to the same NKAE cell concentrations and CD34+ colonies were counted. Data are shown as the mean value ± SEM of three independent experiments

To rule out NKAE cells causing hematological toxicity and destroying hematological stem cells, functional experiments were performed with third party healthy bone marrow mononuclear cells and CD34^+^ hematological precursors from healthy donors. Only 1.7% ± 1.09% of bone marrow mononuclear cells were destroyed at the highest ratio of NKAE:healthy cells (32:1). At lower ratios, cytotoxicity was near 0% (Fig. 7a). Healthy CD34^+^ cells were not destroyed by NKAE cells when clonogenic assays were performed. All of the colonies were able to grow after NKAE cell exposure at all NKAE:CD34^+^ cell ratios (Fig. 7b).

### NKG2D and NKp30 are essential for NKAE cell activity against clonogenic tumor cells

**Fig. 8:**
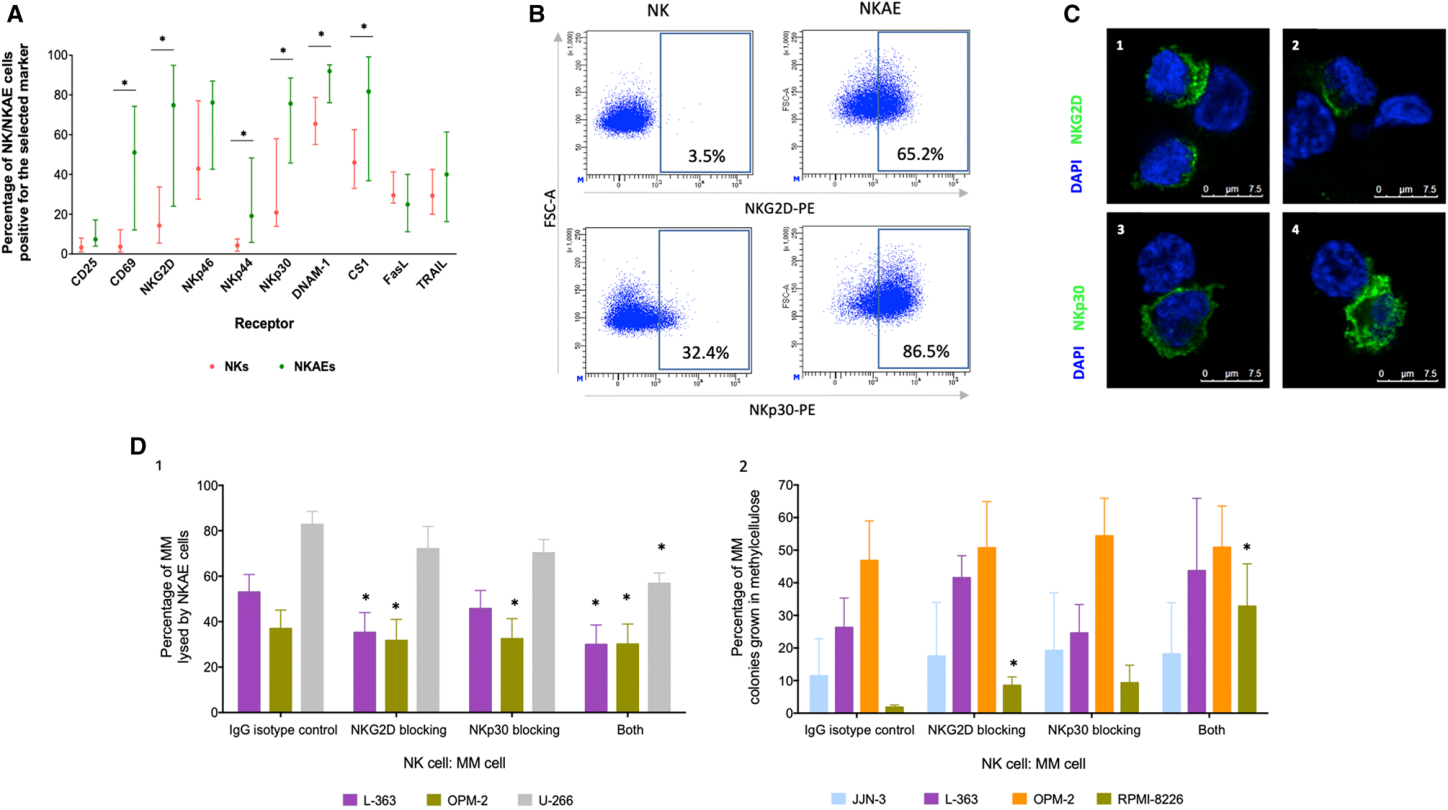
Role of NKG2D and NKp30 in NK cell cytotoxicity against MM. **a** Membrane immunophenotyping of NK cell receptors in NK and NKAE cells from MM patients by flow cytometry. Data are presented as median and IQR (*n* = 14). Paired T test was employed for normally distributed samples and Wilcoxon signed-rank for not normally distributed samples. *p < 0.05 compared to NK cells. **b** Representative dot plot for flow cytometry of NKG2D and NKp30 receptors in NK and NKAE cells. NKAEs exhibited overexpression of these receptors compared to patients’ NK cells before expansion. **c** Representative confocal images of NK-MM aggregates (630X) showing the localization of NKG2D (images 1 and 2) and NKp30 (images 3 and 4) during NKAE cell immune synapses. Alexa Fluor 488 (green) and DAPI (blue, nuclear) staining. **d** NKAE cell activity against MM cells after blocking NKG2D, NKp30, or both in Eu-TDA release assays (1) and colony forming assays (2). NKG2D and NKp30 receptors were blocked in NKAE cells before exposure to cells from different MM cell lines. Results are presented as mean ± SEM. *p < 0.05 NKG2D blocking, NKp30 blocking or both compared to IgG1

The receptors involved in NK cell cytotoxicity were phenotyped in NK cells (*n* = 23) and NKAE cells (*n* = 14) from MM patients. NKAE cells overexpressed all of the analyzed cytotoxicity receptors (i.e., NKp30, NKp44, NKp46, NKG2D, DNAM-1, and SLAMF7), but only the overexpression of NKp30 (*p* = 0.01), NKp44 (*p* = 0.01), NKG2D (*p* = 0.002), and DNAM-1 (*p* = 0.006) was significant (Fig. 8a). In addition, the percentage of positive cells for early activation receptors CD69 (*p* = 0.002) and CD25 increased. Nevertheless, the percentage of positive cells decreased for apoptosis ligand FasL and CD7 receptor, though only the decrease in CD7 was significant (*p* = 0.04).

NKG2D and NKp30 were analyzed in detail, as they were highly overexpressed on NK cells after activation and expansion (Fig. 8b). Fluorescent microscopy revealed that NKG2D localized at the immune synapse formed by NKAE cells with MM cells (Fig. 8c). In contrast, NKp30 was localized all over the cell membrane. Both receptors were blocked with monoclonal antibodies in functional experiments against MM cells and clonogenic cells. A significant reduction in the cytotoxic activity of NKAE cells was observed after receptor blockade in all of the MM cell lines compared to unspecific blockade with IgG1 (Fig. 8d). The most drastic effect occurred with L-363 cells, in which a 17.8% decrease in cytotoxicity was observed.

## Discussion

CSCs have gained increasing attention in cancer research [25, 26]. However, little is known about the characteristics of MM CSCs, partly due to the absence of characteristic surface marker expression [27]. Here, we showed that SP cells could represent the stem cell compartment in MM. CSCs are defined as a group of cells that exhibit resistance to current therapies, so that these therapies target only the non-stem cell compartment and have little or no effect on stem cells [28]. Another important characteristic of CSCs is the self-renewal capacity, which was first described by Drewinko [29]. We have shown that the SP exhibits stem cell properties, such as the clonogenic capacity being an enriched source of ITC in MM cell lines [4]. Moreover, the SP from MM cell lines and primary MM cells from bone marrow exhibited stem cell gene expression, including genes involved in stem cell asymmetric division and stem cell maintenance (Table 1). Studies have demonstrated the overexpression of oncogenes, such as SOX2, NANOG, OCT4, NOTCH, and WNT, in the SP and clonogenic cells [30, 31]. However, as in other studies, the number of overexpressed genes involved in stem cell metabolism represented a small percentage of the total number of genes overexpressed in the SP, which does not reject the hypothesis that the SP is a population of CSCs [32]. The role of snoRNAs in MM is currently not fully known, however it may be involved in the evolution of different stages of the disease [33].

Our data suggest that the expression of stem cell genes could be related to the progression of the disease, as SP cells from the last stages of the disease, such as PCL, exhibited overexpression of a higher number of stem cell metabolism genes compared to MM plasma cells and MGUS plasma cells. The percentage of SP could also be related to disease progression, as PCL SP cells represent 50% of tumor cells [34]. This finding supports the SP representing the stem compartment.

Our studies did not detect the presence of a specific surface marker on CSCs. Some authors have concluded that MM CSCs lack CD138 expression [3, 35]. However, this hypothesis has not been confirmed or has been rejected by other authors who found that CD138^−^ cells correspond to an apoptotic population within the tumor cell population due to a long processing time after taking the sample [36]. If this CD138^−^ population exists, it does not possess tumorigenicity or differential clonogenic properties compared to the CD138^+^ MM population, even in vivo [37, 38].

SP cells exhibit drug resistance, another characteristic of CSCs [2]. Novel anti-myeloma drugs, such as lenalidomide and bortezomib, have been demonstrated to reduce the SP fraction in some MM cell lines. However, their ability to completely eradicate SP cells is not well defined. Bortezomib was able to reduce the SP fraction in two cell lines, without any data from other cell lines or primary samples [5, 12]. Different therapeutic approaches based on combinations of novel drugs with other treatment modalities that could also target SP cells are needed for the complete destruction of SP cells.

In this way, immune-based therapies have gained increasing interest in the last few years and represent a new weapon in the fight against cancer. Previous studies have shown that the immune system is compromised and dysregulated in MM patients, and they postulated that this could be on the basis of the development of the disease [14, 39]. Our results corroborate these findings and show that, within the immune system, NK cells in particular are functionally dysregulated in MM patients, as these cells are not able to destroy MM cells and CSCs in the same way as healthy donors’ NK cells.

Nevertheless, we have demonstrated that NK cells from MM patients can be activated and expanded ex vivo by co-culture with K562-mb15-41BBL cells. The obtained NKAE cells recover cytotoxic activity, even against CSCs [18]. The activity of NK cells against MM has been widely studied. However, studies of NK activity against clonogenic tumor cells in cancer and specifically in MM are limited [23]. Only one study performed by Swift et al., from Armand Keating’s group, showed that NK cells from cell lines were able to destroy clonogenic MM cells. Even knowing that NK cells could be used showing clinical efficacy, this study is the first one showing that primary NK cells specifically destroy the stem cell compartment (tumor cells with drug resistance and clonogenic potential) [40].

Wei et al. demonstrated that immune cells that have been activated by cytokines are capable of destroying stem cell-like cells (characterized by SP detection) from melanoma. In the same way, our in vivo microscopy experiments showed that activated NK cells are capable of destroying SP cells from MM [41].

Ligands of NK cytotoxicity receptors are expressed similarly in SP cells and non-SP cells, suggesting that SP cells could be targeted by NKAE cells in the same way as non-SP cells [42]. We have found that NKAE cells exhibit a highly cytotoxic phenotype, with high expression of cytotoxic receptors, such as NKG2D, and SP cells can be destroyed to a greater extent through the NKG2D receptor, which we have already shown to be essential for NK cell activity against total tumor cells and whose ligands (MICA, MICB, and ULBPs) are overexpressed under stress conditions that occur in cancer, and specifically in MM [43]. This would confirm that NKG2D receptors and ligands are involved in the destruction of SP cells in MM. Although we found downregulation of apoptosis receptors (e.g., TRAIL and FasL) in SP cells, which could be an immune escape mechanism, this pathway does not represent the first mechanism of NK cell activity [21]. Since the blockade of NKG2D and NKp30 does not lead to a complete or near complete abolition of the NKAE cell activity, this raises the need for further in-depth studies of other highly expressed molecules like the co-stimulatory molecule DNAM-1 which has been shown to be related with oxidative stress conditions that may occur in MM [44]. Our results suggest that NKAE cells could represent a new treatment against myeloma, specifically against clonogenic stem cells. However, we have previously shown that better clinical responses are needed [18]. CAR-NK cells have been developed with NK cell lines, although having reduced efficacy because of the need of irradiation before infusion [45]. NKAE cells are autologous cells that lack of related toxicities in the clinical setting and there is no need for irradiation. Here, we hypothetized that NKAE cells could constitute a good approach for CAR transduction to potentiate their anti-myeloma activity. Also, we have found that NKG2D receptor is a key mediator of NKAE cell cytotoxicity. Currently, our group is working on the production of NKG2D-CAR expressing NKAE cells to boost NK cell anti-tumor activity and clinical efficacy.

In summary, the findings added to existing data confirming the drug resistance and self-renewal potential of the SP, laying the foundations to affirm that the SP could represent the stem cell compartment in MM. Therefore, identifying potential therapeutic targets against this population is essential for treatment of MM, not only stopping the disease, but curing it at its origin. On the other hand, we have shown that NK cells are functionally dysregulated in MM patients, and that NKAE cells have the ability to better destroy MM cells, and specifically MM CSCs, than NK cells from MM patients or healthy donors.

### Supplementary Information

Below is the link to the electronic supplementary material.

Supplementary Information (PDF 1376 kb)

## Abbreviations

DCV: Dye Cycle Violet
FBS: Fetal bovine serum
FDR: False discovery rate
GO: Gene Ontology
GSA: Gene-specific analysis
MGUS: Monoclonal gammopathy of undetermined significance
NK: Natural killer
NKAE: Activated and expanded NK
PB: Peripheral blood
PBMC: Peripheral blood mononuclear cells
PCL: Plasma cell leukemia
SP: Side population
snoRNA: Small nucleolar RNA
