# Supplementary material for: Natural killer cells efficiently target multiple myeloma clonogenic tumor cells
Source: Cancer Immunol Immunother. 2021 Mar 10;70(10):2911–24. doi: 10.1007/s00262-021-02901-y (PMC8423695; doi:10.1007/s00262-021-02901-y)
Supplement: Supplementary file 1 — Supplementary Information (PDF 1376 kb) [file 262_2021_2901_MOESM1_ESM.pdf]

Natural killer cells efficiently target multiple myeloma clonogenic tumor cells

Alejandra Leivas [1,2], Ruth M Risueño [3], Alma Guzmán [1], Laura Sánchez-Vega [1,2], Manuel Pérez[4], Diego Megías [4], Lucía Fernández [2], Rafael Alonso [1], Antonio Pérez-Martínez [5], Inmaculada Rapado [1,2], Joaquín Martínez-López [1,2]

[1] Hematology Department, Hospital Universitario 12 de Octubre, Complutense University, instituto de Investigación Sanitaria Hospital 12 de octubre (imas12), Madrid, Spain; [2] H12O-CNIO Haematological Malignancies Clinical Research Unit, Spanish National Cancer Research Center, Madrid, Spain; [3] Leukemia stem cell group, Josep Carreras Leukaemia research Institute, Barcelona, Spain; [4] Confocal Mycroscopy Unit, Spanish National Cancer Research Center, Madrid, Spain; [5] Pediatric Hemato-Oncology Department, Hospital La Paz, Madrid, Spain;

Supplementary tables

Antigen	Clone	Fluorochrome	Source	Catalog number
CD3	UCHT1	PE/Cy7	Biolegend	351304
CD4	RPA-T4	APC/Cy7	Biolegend	300518
CD7	CD7-6B7	FITC	Biolegend	343104
CD16	3G8	APC/Cy7	BD Pharmingen	557758
CD19	SJ25C1	PE	Biolegend	363004
CD25	BC96	FITC	Biolegend	302604
CD31	WM59	FITC	Biolegend	301104
CD45	HI30	FITC	Biolegend	304006
CD56 (NCAM)	HCD56	APC	Biolegend	318310
CD127 (IL-7Rα)	A019D5	PE	Biolegend	351304
CD69	FN50	PE	Biolegend	310906
CD178 (FasL)	NOK-1	PE	Biolegend	306407
CD253 (TRAIL)	RIK-2	PE	Biolegend	308206
DNAM-1 (CD226)	TX25	FITC	Biolegend	337104
NKG2A (CD159a)	#131411	PE	R&D Systems	FAB1059P
NKG2D (CD314)	1D11	PE	Biolegend	320806
NKp30	AF29-4D12	PE	Miltenyi	130-092-483
NKp44	Z231	PE	IOtest	PNIM3710
NKp46	9E2	PE	Biolegend	331908
PROPIDIUM IODURE			Sigma Aldrich	P4864

Supplementary Table S1. **Antibodies used in flow cytometry.** FITC, fluorescein isothiocyanate; PE, phycoerythrin; PE/Cy7, tandem constituted by phycoerythrin and cyanin 7; APC, allophycocyanin; APC/Cy7, tandem constituted by allophycocyanin and cyanin 7.

Patient	Age, years	Sex	Percentage of side population	Type	Bone marrow infiltration	Situation when taking sample
01	56	M	1.05%	IgG κ	98%	Newly diagnosed
02	72	F	3.69%	IgG κ	53%	Biological progression
03	81	M	0.8%	IgA κ	1%	MGUS
04	64	F	0.3%	IgG λ	38%	Newly diagnosed
05	84	M	2.3%	IgG λ	2.1%	Stable disease

Supplementary Table S2. **Clinical characteristics of the analyzed multiple myeloma patients.** Samples from multiple myeloma patients were used for side population studies and phenotyping.

Cell line	SP	SP + RES	Bone marrow samples	SP	SP + RES
JJN-3	1.3%	0.9%	1	1.9%	0.2%
L-363	2.1%	0.2%	2	1.48%	0.00%
MM.1S	0.2%	0.2%	3	2.3%	0.2%
NCI-H929	0.834%	0.007%	4	11.4%	1.3%
OPM-2	0.6%	0.1%	5	0.5%	0.4%
RPMI-8226	4.5%	0.6%	6	0.2%	0.2%
SK-MM-2	0.136%	0.007%	7	1.9%	1.2%
U-266	2.2%	0.1%	8	0.9%	0.6%

Supplementary Table S3. Multiple myeloma side population (SP) distribution. Data are given as the percentages of SP in multiple myeloma cell lines and bone marrow samples. Percentages after incubation with reserpine (RES) are also shown for each analyzed sample. Primary patient samples were anonymized.

Supplementary figures

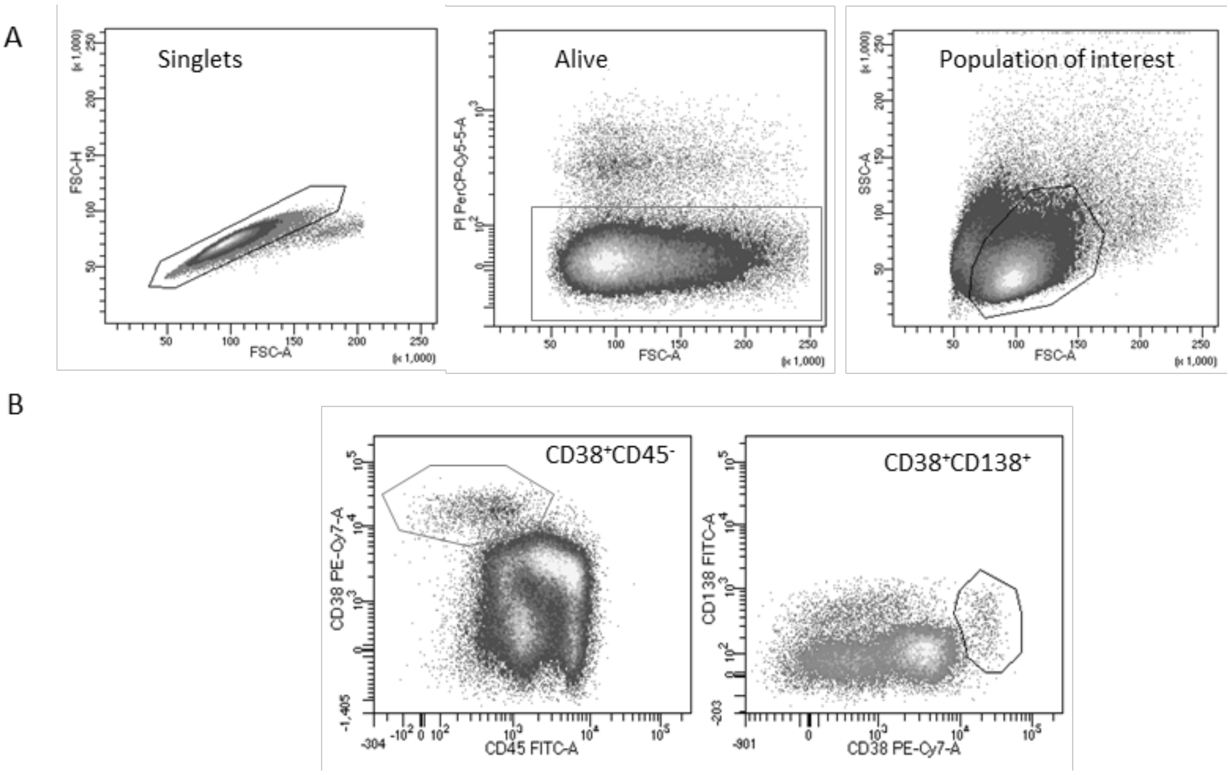

Supplementary Figure S1. Flow cytometry strategy for identification of the multiple myeloma side population. (A) Representative dot plots. Doublets and dead cells were excluded before gating the cell population of interest. **(B)** In multiple myeloma bone marrow samples, pathological plasma cells were gated based on the expression of CD138 and CD38 and lower expression of CD45.

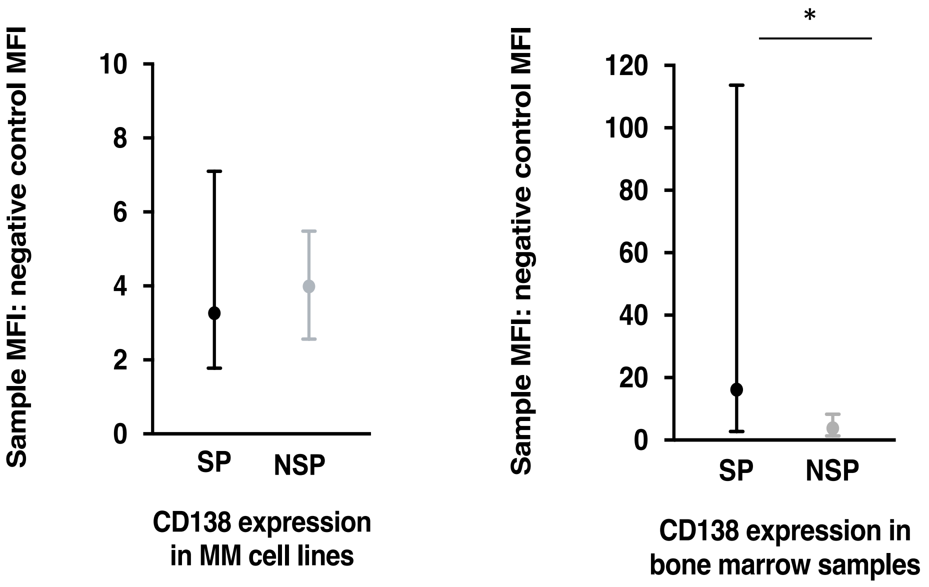

Supplementary Figure S2. CD138 expression in SP and NSP cells. C138 expression on SP and NSP cells was evaluated by flow cytometry. Eight MM cell lines and five fresh primary bone marrow samples were analyzed. Data are presented as median and IQR. *$p < 0.05$ compared to NSP cells.

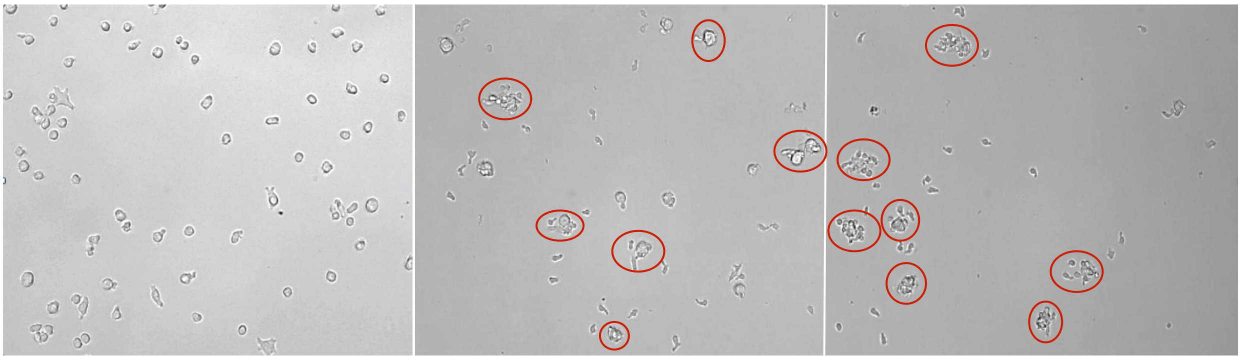

Supplementary Figure S3. Natural killer (NK) cell cytotoxicity against multiple myeloma (MM) bulk and side population cells. Representative image of time-lapse microscopy of NK cell synapses with MM. NK cells from MM patient vs. MM cells (left), NKAE from the same patient vs. MM cells (center), and NKAE cells vs. SP cells (right). NK cell-MM cell aggregates are indicated by red circles.
